# Orientation-invariance of individual differences in three face processing tasks

**DOI:** 10.1098/rsos.181350

**Published:** 2019-01-09

**Authors:** G. Meinhardt, B. Meinhardt-Injac, M. Persike

**Affiliations:** Department of Psychology, Johannes Gutenberg University, Mainz, Germany

**Keywords:** face inversion effect, face-specific processing, individual differences, selective attention

## Abstract

Numerous studies have reported impairments in perception and recognition, and, particularly, in part-integration of faces following picture-plane inversion. Whether these findings support the notion that inversion changes face processing qualitatively remains a topic of debate. To examine whether associations and dissociations of the human face processing ability depend on stimulus orientation, we measured face recognition with the Cambridge Face Memory Test (CFMT), along with experimental tests of face perception and selective attention to faces and non-face objects in a sample of 314 participants. Results showed strong inversion effects for all face-related tasks, and modest ones for non-face objects. Individual differences analysis revealed that the CFMT shared common variance with face perception and face-selective attention, however, independent of orientation. Regardless of whether predictor and criterion had same or different orientation, face recognition was best predicted by the same test battery. Principal component decomposition revealed a common factor for face recognition and face perception, a second common factor for face recognition and face-selective attention, and two unique factors. The patterns of factor loadings were nearly identical for upright and inverted presentation. These results indicate orientation-invariance of common variance in three domains of face processing. Since inversion impaired performance, but did not affect domain-related associations and dissociations, the findings suggest process-specific but orientation-general mechanisms. Specific limitations by constraints of individual differences analysis and test selection are discussed.

## 1. Introduction

In the last two decades, the face inversion effect has received much attention across all disciplines of face perception research. However, opposing views persist about its implications for underlying face processing mechanisms as well as its potential neural underpinnings [1–4]. In a widely accepted view, the strong impairment in face recognition and identification tasks with inverted stimuli reflects qualitative changes in facial information processing. Several lines of evidence support a qualitative account of the face inversion effect. First, and phenomenologically striking, the impression of grotesqueness in face pictures with rotated eyes and mouth (‘Thatcher illusion’; [5,6]) is greatly attenuated in upside-down faces. This led to the conjecture that spatial-configural information processing is intact in the usual upright orientation, but disrupted by inversion [7–9]. Moreover, different measures of holistic face processing, all indicating this mode in the upright orientation, suggest that the degree of part integration is notably attenuated when faces are inverted [10–12]. Results from neuroimaging studies suggest that inverted faces engage face-tuned areas in the ventral stream, but also areas that preferentially respond to non-face objects, which might indicate a change of the processing route [4,13]. This was corroborated by the observation that patients with object agnosia could handle upright faces well, but had problems with inverted faces, suggesting that their efficient processing requires more than intact face processing mechanisms [14]. Further, the N170, an event-related potential from occipito-temporal electrodes, shows a significant delay for inverted compared to upright faces [15,16]. These results might indicate involvement of different mechanisms for upright and inverted faces.

On the other hand, evidence accumulates for the claim that upright and inverted faces rely on the same, or tightly overlapping sets of visual cues, and are processed by the same mechanisms, but with reduced efficiency for upside-down presentation. In line with this view, it was shown that there is no stringent distinction of ‘featural’ and ‘configural’ processing in terms of the inversion effect [3,17,18]. Measuring activation in face-selective brain areas showed strong object category selectivity rather than orientation selectivity [18,19]. Response classification methods revealed usage of the same cues in face discrimination tasks, but with less efficiency for inverted faces [20]. Facial cue summation experiments showed that inverted features were integrated in a less efficient manner than upright features, but according to the same rules [21]. Readdressing the spatial frequency tuning of upright and inverted face identification, a recent study found no evidence for orientation-related changes in spatial frequency tuning [22]. Critical evaluation of the results obtained in studies measuring whether face parts can be attended independently (see above) showed that the influence of non-attended face parts was diminished, but not abolished when faces were inverted (e.g. [11, fig. 4], [12, fig. 5]), indicating that results are not at odds with a quantitative account of the face inversion effect (see also [23] for further evidence). Moreover, neuroimaging studies do not provide unique evidence in favour of qualitative changes. While it is corroborated that face inversion engages additional processing regimes in face and non-face object sensitive areas, such as the occipital face-selective region (OFA), the superior temporal sulcus (fSTS) or the lateral occipital complex (LOC), the functional relevance of face-related activity in these areas remains unclear. Activity which varied in close correlation to the behavioural inversion effect was first found only in the fusiform face area (FFA) [13], while a later study also reported involvement of the OFA [24], suggesting the functional relevance of the same face-tuned core-units for processing both upright and inverted stimuli.

In another recent psychophysical study, sensitivity to part-based and relational manipulations of faces and houses was measured in a sample of 77 observers [25]. A correlation analysis was performed to reveal potential common category-specific or process-specific (spacing-tuned or feature-tuned) processing. The authors replicated the finding of similar inversion effects for both types of image manipulations, and found evidence for two domain-general, but process-specific factors (see [25], table 3), each explaining about 30% of variance. Relational, or ‘spacing’, conditions had high loadings only on the first factor and part-based conditions only on the second. Moreover, loadings were equally high for upright and inverted presentation. These findings suggest shared processing routes for upright and inverted stimuli, with no changes by inversion. Quite consistently, common variance was found to be process-specific but unspecific for orientation.^1^

A recent study on the developmental trajectory of part-based and configural object recognition across adolescence also found evidence for distinct processes with specific developmental trajectories [26]. Part-based processing was found to saturate early in adolescence, reaching a plateau after 11–12 years of age, while configural processing steadily improved well into young adulthood (see [26], figure 2). Inversion effects were found for both kinds of processing, while the distinct, process-specific trajectories had exactly the same temporal course in either orientation. In line with the findings from individual differences analysis [25], these results from cross-sectional data suggest process-specific but orientation-invariant routes.

These results point to a potential orientation-invariance principle for common variance structures that could be validated by individual differences data. There is growing interest in this approach for resolving controversial issues in the face processing literature, since it offers access to patterns of associations and dissociations among different face processing abilities, which are usually ignored in experimental studies which focus on group-level analysis [27]. Predicting face recognition, measured with representative tests like the Cambridge Face Memory Test (CFMT) [28,29], by cognitive and perceptual abilities has raised considerable attention in the past years [27,30,31]. Face recognition is a highly heritable [29,32], long-lasting developing [33] and specific ability, which is almost independent of general intelligence [31,34,35], non-face memory ability [29], and general object recognition ability [36,37]. It is also a complex ability, comprising face perception and encoding, face memory and cognitive capabilities to infer face identity from unknown views, distorted images, varying external features or age-related changes [28,38,39]. Accordingly, it was found that face recognition was associated with face perception and long-term face memory [29,39,40]. Surprisingly, it appeared to be just weakly associated [41,42], or even dissociated from holistic face perception, indexed by derived measures used in varieties of the composite face paradigm [43,44]. Difference measures of part-whole integration showed modest correlation with face recognition, while larger correlations were obtained with regression-based methods [45]. However, results of a recent study [46] showed that part and whole performance correlated nearly perfectly, casting doubts on the construct validity of the part-whole test. The suitability of different holistic processing measures for predicting face recognition ability is currently under debate (e.g. [47]).^2^

In the present study, we focused on the associations and dissociations among face recognition, face perception and object-related attention to faces (face-selective attention) as three key domains of human face processing. Albeit the literature on individual differences in the face recognition ability is steadily growing [31], there were hitherto no attempts to reveal how correlations with face recognition depend on stimulus orientation. This study is a first attempt to contribute to this question. To assess face perception ability, we used a paradigm that allowed us to measure how precisely observers were able to judge identity of just the inner face parts, ignoring the external facial surround (context congruency paradigm, [12]).^3^ Object-related attention tasks were administered with faces and non-face objects to study whether the potential link between object-related attention and face recognition is face-specific. We administered a comprehensive test battery, including only tests with reasonably good internal consistency, to a broad sample of 314 participants, and measured all test conditions with upright and inverted stimuli. Principal component and multiple regression analysis revealed distinct process-specific pathways for face-related tasks, two of them shared with the face recognition ability. Surprisingly, the patterns of associations and dissociations within and across tests were the same for upright and inverted stimuli, indicating orientation-invariance of correlation structures. These findings indicate that orientation-general mechanisms underlie stimulus processing in shared processing routes for the studied face-related tasks.

## 2. Material and methods

### 2.1. Study outline

Three experimental tests were administered: one for face recognition, one for face perception and one for selective attention. All tests were administered in one 90 min session. The order of the tests was counterbalanced across participants. Test were separated by a 10 min break. Three hundred and fourteen observers participated in the study. All participants were undergraduate students of the Johannes Gutenberg University Mainz. The age range was 17–37 years, mean age = 22.8 years, standard deviation = 3.4 years, 223 were female. The participants had normal or corrected to normal vision, using corrective lenses in the latter case. All participants received a global information about the tests and the abilities being assessed with the test battery. They were given a gratification of €30 for participation.

### 2.2. Apparatus

All experimental tests were computer-administrated, executed with Inquisit runtime units. Stimuli were displayed on NEC Spectra View 2090 TFT displays in 1600 × 1200 resolution at a refresh rate of 60 Hz. Screen mean luminance was 100 cd m^−2^ at a Michelson contrast of = 0.98. No gamma correction was used. The room was darkened so that the ambient illumination matched that of the screen. Stimuli were viewed binocularly at a distance of 70 cm. Participants used a distance marker but no chin rest throughout all experiments. Participants responded by pressing a button on an external key-pad. No feedback about responses was provided in any of the three tests.

### 2.3. Cambridge Face Memory Test

The Cambridge Face Memory Test (CFMT) [28] was developed to study face identity recognition, including generalization over viewpoint and image distortion. Six different target faces and 46 distracter faces are used. The participant is asked to memorize six target faces for 20 s (encoding). Afterwards, sequences of six face images are presented, one of them a target face. In the first 18 sequences, the target item is identical with an encoded item. Over the following 30 sequences, the identity of the target and the encoded item stays the same, but the target item shows the encoded item in a different view (left or right profile). The final 24 items show the target items in a different view and distorted by noise. All test sequences were conducted with upright and inverted presentation of the stimuli, organized in coherent blocks. Block order was counterbalanced across participants. The total test score is the number of correctly identified target items in all of the 72 sequences. With an additional learning phase, the test takes about 15 min, adding up to a total of 30 min for both upright and inverted conditions. Detailed information on test construction can be found in [28]. The authors provide free access to the test material for scientific purposes.

### 2.4. Context congruency paradigm

**Figure 1.**
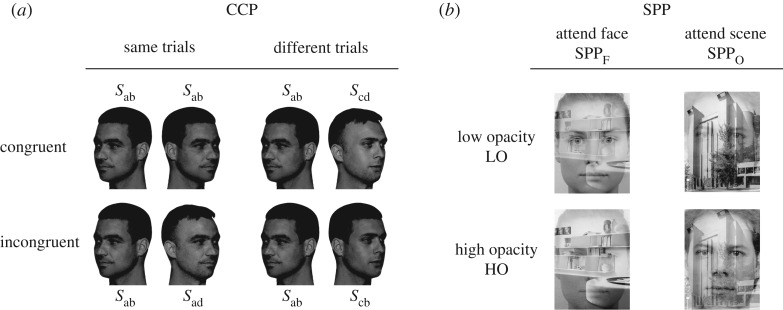
The context congruency paradigm (*a*) and the superposition paradigm (*b*). (*a*) Illustrates congruent (total sameness and difference) and incongruent (part-based sameness and difference) trials of the CCP in the framework of the complete design. The congruency relation refers to inner face part matching in the context of external feature surround. The letters a–d refer to face identities, the first index denotes internal features, the second external features. (*b*) Shows stimulus examples for the superposition paradigm for attending faces while ignoring scenes, SPP_F_, and, vice versa, SPP_O_. The non-attended stimuli were realized with high (65%, HO) and low (35%, LO) values of opacity to construct conditions with high and low attentional competition among faces and scenes.

The context congruency paradigm (CCP) was developed to measure the interaction among external and internal facial features in face perception, thus indicating the degree of joint processing of inner and outer face parts [12,49]. High internal consistency was verified for all test conditions (see Methods). In the variant used here, participants judged the identity of the internal facial features of two successively shown faces, one in left-hand sided and one in right-hand sided $\frac{3}{4}$ view. The complete design [50] was used to vary the congruency relation of internal and external features (figure 1). Congruent contexts (CC) were defined as total agreement of faces in ‘same’ trials and total disagreement in ‘different’ trials. Incongruent contexts (IC) were defined as part-based agreement/disagreement. Specifically, in incongruent-same trials the internal features of a face pair agreed, while the external features disagreed. In incongruent-different trials, the inner face parts disagreed while the external parts agreed. Responding to whole face agreement/disagreement is beneficial in congruent trials, but leads to chance performance in incongruent trials (see [12] for detailed descriptions).

#### 2.4.1. Stimuli

Photographs of four male face models taken in a professional photo studio were used as templates for stimulus construction. The photographs were converted to 8 bit greyscale pictures. Face models had similar haircut, face geometry and overall type appearance. As a scheme for stimulus construction, we used a 4 × 4 composite face matrix with line index referring to internal features and column index referring to external features, such that an entry *S*_*ij*_ denotes a face with internal features of face *i* and external features of face *j*. Before placing the internal features of face *j* onto face *i*, mean grey level and contrast of the internal features of face *j* were adjusted to the values of the internal features of face *i*. As a result, internal features were smoothly integrated into the external feature surround. Thus, we obtained 16 natural looking composite faces, to be used for same-congruent trials. For each composite it is possible to combine in nine different ways to form complete sets of trials within the complete design [50]. Hence, using 16 ‘same’ and 16 ‘different’ trials warranted that there was no repetition of the same stimulus pair, and that each observer responded to an individual choice of trial selections. Stimulus size was 300 × 400 pixels (width × height), which corresponded to 7.0 × 9.7 cm, or 5.7° × 7.9° visual angle. For each stimulus, an individual mask was constructed from randomly ordered 5 × 5 pixel blocks of the stimulus image. Masks subtended 450 × 600 pixels (width × height). (See [12] for more details.)

#### 2.4.2. Procedure

Participants were informed that two face images would be presented successively, and that the identity of the inner face parts had to be judged. They were also told that the external face parts could vary or be identical and were irrelevant for the identity judgement. The structure of a trial was: fixation mark (300 ms)–blank (100 ms)–first face stimulus (433 ms)–mask (350 ms)–blank (200 ms)–second face stimulus (433 ms)–mask (350 ms)–blank frame until response. The centre positions of each of the two face images were shifted by 20 pixels away from the centre in random direction to preclude the same image parts being focused. In half of the trials, the first face image was in left-hand sided view, and in the other half in right-hand sided view. Further, both face images of a trial sequence were presented upright, or rotated by 180° (inverted). The two congruency conditions (congruent, incongruent), two orientations (upright, inverted), two trial types (same, different) and 16 replications of condition resulted in 128 trials, which were ordered randomly. The proportion of correct judgements, calculated from both correct ‘same’ and correct ‘different’ judgements, was measured. Participants were made familiar with the task with 16 randomly selected practice trials. The experimental test took about 20 min.

### 2.5. Superposition paradigm

The superposition paradigm (SPP) was developed to study attentional competition among coherent sensory inputs (objects) presented simultaneously by spatial overlap [51,52]. As in [52], we studied attentional competition among faces and scenes in two categorization tasks. In the face categorization task (SPP_F_), a face image was overlayed in transparency with a scene image of the same size, and the participants judged the gender of the face. In the scene categorization task (SPP_O_), they judged whether the scene image depicted was an interior or exterior scene. Two levels of opacity of the irrelevant, to be ignored image were used: 35% (low opacity, LO) and 65% (high opacity, HO), (figure 1). The LO condition warrants high salience of target objects and low levels of competition, while there is no salience of target objects and a high level of attentional competition in the HO condition. Composite face+scene images were created by combining pictures from a database with 18 male and 18 female faces and 18 interior and 18 exterior scenes. Stimulus picture dimensions were the same as in the CCP. Gender and scene category were varied orthogonally to create composites, i.e. a face of a given gender was paired with both interior and exterior scenes, and vice versa. The design comprised two tasks (gender/scene categorization), two stimulus orientations (upright/inverted) and two levels of opacity (LO/HO). Each of these eight conditions was executed with 32 replications, resulting in 128 trials for each categorization task. The proportion of correct classifications, calculated from both response alternatives in each test, was measured.

Participants were made familiar with each task by going through five randomly selected probe trials. A trial started with presentation of a central fixation mark for 1 s, which was replaced by the compound image for 2.5 s. Response triggered presentation of the next trial, with an inter-trial period of 2 s. The order of the two categorization tasks was counterbalanced across observers. Each categorization task took about 15 min testing time.

### 2.6. Data analysis

In total, 14 performance indices, seven for upright and seven for inverted presentation, were obtained from the three experimental tests. All indicated the proportion of correct judgements. The CFMT yielded two sumscore indices, one for each orientation. The CCP yielded four indices, reflecting performance in congruent and incongruent condition for each stimulus orientation. Each categorization task of SPP again yielded four indices, reflecting performance for both opacity levels for upright and inverted presentation. To ensure that the correlation analyses were not biased by outliers, we conducted multivariate outlier analysis based on the squared Mahalanobis distance measure, Δ^2^ ([53, pp. 177]). To clear for multivariate outliers, we calculated the distribution of Δ^2^ values, and removed all cases with values beyond the third quartile plus 1.5 units of inter-quartile range (Tukey’s method, [54]). This method identified 16 outliers, which were excluded from data analysis. Thus, *N* = 298 of the 314 cases remained in the final sample. Inspection of the (142)=91 bivariate regression plots verified that correlations were not biased by single observations. The proportion correct measures, *P*_*c*_, were analysed with ANOVA methods for group-level analyses. Additionally, inversion effects (IEs), congruency effects for the CCP and opacity effects for the SPP were calculated with paired *t*-tests. Cohen’s *d* was calculated for all tests to provide the same effect size measure for all effects. Statistica 13.0 software was used for all ANOVA-based testing, principal component analysis and multiple regression methods.

### 2.7. Assessment of test reliability

Internal consistencies for all tests were assessed by computing Cronbach’s *α*, which is based on inter-item correlations [55]. Test trials were treated as items. Overall test reliability was calculated by applying the Spearman–Brown test length factor formula to the single test parts. Table 1 shows the results for the CFMT and table 2 summarizes the results for the CCP and the SPP. The CFMT showed high overall internal consistency, exactly in line with the estimate reported by the test authors from a sample of 389 participants [29]. Internal consistency was reduced in inverted face presentation, mostly due to a noticeable loss of homogeneity in the third test part with external noise. Similarly, the CCP exhibited high overall internal consistency, which was larger for congruent (full face agreement/disagreement) than for incongruent trials (partial agreement/disagreement). Internal consistency of the SPP was acceptable, higher for SPP_O_ compared with SPP_F_, which reflected stronger item-heterogeneity in the gender categorization task. Since only one test suffered from reduced reliability we calculated across-test correlations without corrections for attenuation.

**Table 1.** Internal consistency of the CFMT. The table shows Cronbach’s *α* calculated from the inter-trial correlations in the three test parts, and Spearman–Brown based reliability estimate for the complete test, *r*_*tt*_.

	upright			inverted		
	T1	T2	T3	T1	T2	T3
*α*	0.69	0.81	0.78	0.58	0.56	0.25
*r*_*tt*_	0.88			0.70

**Table 2.** Internal consistency of the CCP and the SPP. The table shows Cronbach’s *α* and Spearman–Brown reliability estimates for the test conditions, *r*_*tt*_.

	upright				inverted			
CCP	CC		IC		CC		IC	
	same	diff	same	diff	same	diff	same	diff
*α*	0.80	0.80	0.70	0.68	0.75	0.68	0.60	0.66
*r*_*tt*_	0.89		0.82		0.83		0.78	

SPP	face		scene		face		scene	
	HO	LO	HO	LO	HO	LO	HO	LO
*α*	0.33	0.29	0.61	0.65	0.39	0.44	0.55	0.49
*r*_*tt*_	0.47		0.77		0.58		0.69	

## 3. Results

### 3.1. Single test results

**Figure 2.**
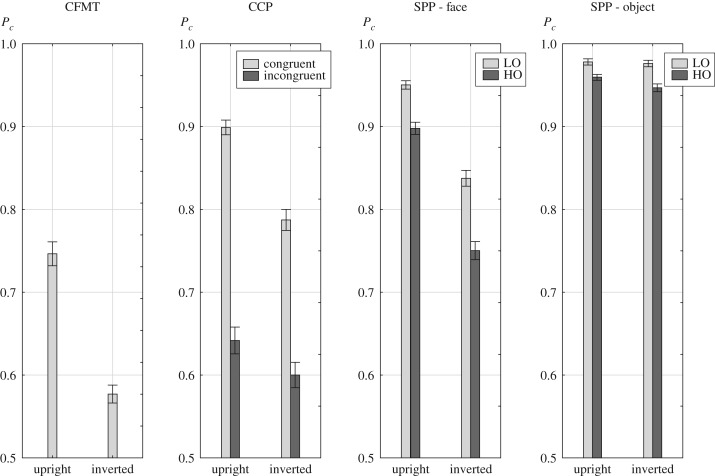
Mean proportion correct rates, *P_c_*, for all test conditions. The error bars denote the 95% confidence intervals of the means.

Figure 2 shows the mean accuracy data for all three tests, and table 3 shows inversion effects.

**Table 3.** Inversion effects in the CFMT, the CCP and the SPP. The table shows the difference measure, IE, its standard error, *t* − statistic, significance level, *p*, and Cohen’s *d*. All *t*-tests had 297 degrees of freedom.

test	condition	IE	*s*_*e*_	*t*	*p*	*d*
CFMT	all	0.169	0.007	25.7	<0.001	1.49
CCP	congruent	0.112	0.006	19.7	<0.001	1.14
	incongruent	0.042	0.007	5.7	<0.001	0.33
SPP	face-LO	0.113	0.005	22.6	<0.001	1.31
	face-HO	0.148	0.006	26.5	<0.001	1.53
	scene-LO	0.001	0.002	0.1	0.894	0.01
	scene-HO	0.013	0.002	5.7	<0.001	0.33

**Figure 3.**
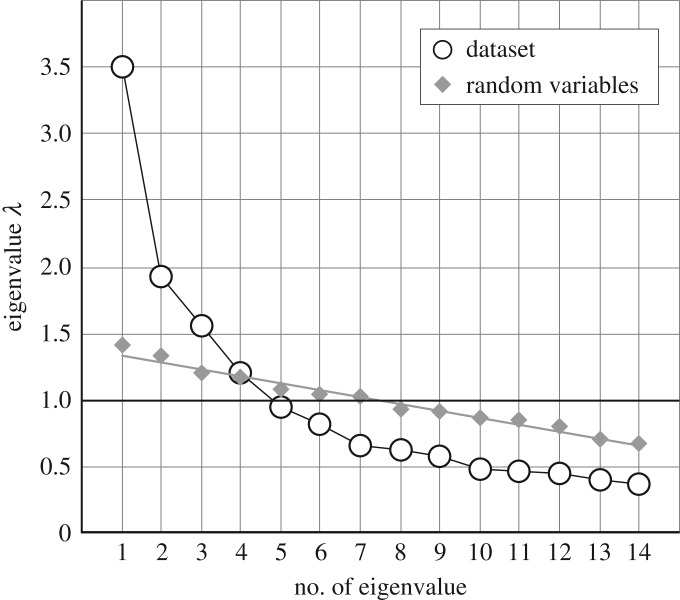
Factor scree plot for the principal component decomposition of all tests (open circles). Grey diamonds indicate the Eigenvalues of normally distributed and independent random variables with the same sample size as the dataset.

In the CFMT, an average accuracy based on the total test score of *P*_*c*_ = 0.746 was achieved in upright and *P*_*c*_ = 0.577 in inverted presentation, which fairly well agreed with the data of the test authors, who obtained rates of *P*_*c*_ = 0.804 (upright) and *P*_*c*_ = 0.584 (inverted) in a sample of 50 college students [28, p. 580]. The inversion effect was large, and reached an effect size of *d* = 1.5 (table 3). In the CCP, there were significant effects of Congruency [*F*_1,297_ = 925.9, *p* < 0.001, *η*_*p*_ = 0.76], Orientation [*F*_1,297_ = 259.9, *p* < 0.001, *η*_*p*_ = 0.47] and a significant Orientation × Congruency interaction [*F*_1,297_ = 59.5, *p* < 0.001, *η*_*p*_ = 0.17]. This interaction indicated a larger congruency effect in upright compared to inverted presentation. However, congruency effects in both orientations were substantial, reaching large effect sizes (upright: Δ = 0.26, *t*_297_ = 30.6, *p* < 0.001, *d* = 1.77; inverted: Δ = 0.19, *t*_297_ = 21.3, *p* < 0.001, *d* = 1.24), while inversion effects were large in congruent trials, but had modest effect size in incongruent trials (table 3).

In the SPP, there were significant effects of Stimulus [*F*_1,297_ = 1166, *p* < 0.001, *η*_*p*_ = 0.80], Opacity [*F*_1,297_ = 790.1, *p* < 0.001, *η*_*p*_ = 0.73] and Orientation [*F*_1,297_ = 1039, *p* < 0.001, *η*_*p*_ = 0.78]. Further, all 2-way interactions were highly significant [Opacity × Orientation: *F*_1,297_ = 40.2, *p* < 0.001, *η*_*p*_ = 0.12; Stimulus × Opacity: *F*_1,297_ = 68.2, *p* < 0.001, *η*_*p*_ = 0.19; Stimulus × Orientation: *F*_1,297_ = 896.3, *p* < 0.001, *η*_*p*_ = 0.75], and also the Stimulus × Opacity × Orientation interaction was significant [*F*_1,297_ = 7.9, *p* < 0.01, *η*_*p*_ = 0.03]. The 2-way interactions involving opacity reflected significantly larger opacity effects in inverted compared to upright presentation, and significantly larger opacity effects for attending scenes (*d* = 1.06) than for attending faces (*d* = 0.82).^4^ Inversion effects with large effect sizes were reached for attending faces in both opacity conditions. For attending scenes, an inversion effect was absent in the LO condition, but there was a modest inversion effect in the HO condition (table 3).

### 3.2. Discussion of single test results

The CFMT, the CCP in congruent conditions and the SPP for attending faces all yielded strong inversion effects (*d* > 1) (table 3). On the other hand, the CCP in incongruent condition and the SPP for attending scenes exhibited moderate (HO), or even lacking (LO) inversion effects, which were clearly distinguished from the aforementioned large inversion effects in terms of effect size. The expected strong effects of congruency (CCP) and opacity (SPP) at the group level verified the experimental paradigms. Larger opacity effects for scenes compared to faces showed that faces were more robust than scenes in object-based attentional competition [52].

Performance in the congruent condition of the CCP suffered notably from inversion. However, the congruency effect in inverted presentation was still quite substantial (*d* > 1, see figure 2. See also [23] for similar observations in the composite paradigm). This shows that contextual interference from external features was diminished but not abolished for inverted faces. Likewise, we found substantial opacity effects of the non-face masker for the SPP_F_ in both orientations, which indicates that the nature of attentional competition was not altered by inversion.

### 3.3. Predictions for individual differences

Several lines of evidence suggest a common route for upright and inverted stimuli, while there is also evidence for orientation-dependent processing (see Introduction). A strong claim of orientation-invariance would consider only orientation-general mechanisms. This claim has two implications. First, it implies that performance in the same test condition is solely explained by a common mechanism for upright and inverted stimuli, up to spurious error variance. Second, it implies that the correlation structures of a test battery are the same for either orientation.

A weaker claim of orientation-invariance would consider that both orientation-general and orientation-specific mechanisms handle a given test condition, but would postulate that only orientation-general mechanisms underlie shared processing within and across tests. This would imply (i) a strong orientation-general variance component in each test condition and (ii) same correlation structures of a test battery for either orientation.

### 3.4. Strategy of testing predictions for individual differences

The strong claim of orientation-invariance can be tested using the bivariate (zero-order) correlations among both orientations for the same test conditions. If the correlation among both orientations, *r*, is close to the maximum possible correlation, which is limited by the geometric mean of the reliabilities for either orientation, $r_{\max}=\sqrt{r_{tt}r_{t^{\prime }t^{\prime }}}$, this would indicate just a common route for both orientations, since there is no reliable unexplained variance left.

A redundant test strategy, which analyses the data from different angles, is recommended for testing whether the correlation structure of the test battery is orientation-invariant. Since face recognition is a complex ability (see Introduction), we may predict the CFMT by the remainder tests, and probe whether the results for regression weights and explained variance depend on stimulus orientation. Orientation-invariance would be concluded if the best predictor battery is the same, irrespective of whether the predictors have same or different orientation than the criterion. Another way of testing orientation-invariance of correlations is decomposing the correlation matrix of all test conditions into principal components. In a factor solution for the common variance components,^5^ we should observe that, if a test has significant factor loadings, the loadings should be equally strong for either orientation. The results of multiple regression analysis are validated by the factor solution if it is shown that the CFMT and its valid predictors load on the same factors, and equally strong for upright and inverted stimuli.^6^

### 3.5. Results of individual differences analyses

#### 3.5.1. Zero-order (bivariate) correlations

We calculated bivariate product-moment correlations among all test conditions. Table 4 shows the results. With *N* = 298 cases, the critical correlations for significance levels of α=0.1%, α=1% and α=5%, respectively, are $r_{\left(0.1\text{\%}\right)}=0.19$, $r_{\left(1\text{\%}\right)}=0.15$ and $r_{\left(5\text{\%}\right)}=0.11$. In the table, the correlations larger than $r_{\left(0.1\text{\%}\right)}$ are printed boldface.

**Table 4.** Pearson product-moment correlations of the CFMT, the CCP, the SPP_F_ and the SPP_O_ for all seven test conditions and both orientations. Correlation coefficients larger than the critical correlation $r_{\left(0.1\text{\%}\right)}=0.19$ are printed boldface. Coefficients for the same test condition but across orientations are underlined.

test			CFMT		CCP				SPP_F_				SPP_O_		
Cond			—	—	CC	CC	IC	IC	LO	LO	HO	HO	LO	LO	HO
		orient	upr	inv	upr	inv	upr	inv	upr	inv	upr	inv	upr	inv	upr
*CFMT*		inv	**0.50**												
CCP	CC	upr	**0.28**	**0.35**											
CCP	CC	inv	**0.27**	**0.27**	**0.52**										
CCP	IC	upr	0.10	0.13	**0.23**	**0.19**									
CCP	IC	inv	0.13	0.12	**0.26**	**0.25**	**0.58**								
SPP_F_	LO	upr	0.19	0.12	−0.01	0.05	0.07	0.17							
SPP_F_	LO	inv	0.16	**0.27**	0.15	0.14	−0.04	0.08	**0.22**						
SPP_F_	HO	upr	**0.22**	**0.24**	0.15	0.16	0.05	0.12	**0.23**	**0.44**					
SPP_F_	HO	inv	**0.21**	**0.27**	0.14	0.12	0.13	**0.20**	0.17	**0.40**	**0.33**				
SPP_O_	LO	upr	0.04	0.06	0.05	0.10	0.06	0.08	0.08	0.13	0.14	0.02			
SPP_O_	LO	inv	0.11	0.03	0.10	0.17	0.12	0.17	0.13	0.11	**0.20**	0.13	**0.44**		
SPP_O_	HO	upr	0.11	0.17	0.10	0.08	0.15	0.15	**0.26**	**0.25**	**0.25**	**0.25**	**0.48**	**0.50**	
SPP_O_	HO	inv	0.04	0.13	0.11	0.08	0.11	0.08	0.11	**0.20**	**0.20**	**0.20**	**0.41**	**0.44**	**0.50**

There are several crucial observations concerning the pattern of bivariate correlations. First, there were strong and significant correlations among conditions within the same tests (diagonal quadrants of table 4). These correlations were similar for upright and inverted presentation. They tended to be highest for the same condition, measured across orientations (see underlined values in table 4). Second, there were selective correlations between tests. Whenever between-test conditions correlated significantly, the correlation magnitude was alike in both orientations, and across them, reflected by quite homogeneous blocks of four correlations in table 4. Looking at the bivariate correlations of the CFMT shows that there were significant correlations with the CCP in congruent condition (both orientations), but not in the incongruent condition. Further, the CFMT correlated most substantially with the SPP_F_ in the high opacity condition (both orientations), and weaker for low opacity of the masker. With just one exception, there were no highly significant correlations with SPP_O_ conditions in the low opacity condition, while there were correlations among SPP_F_ and SPP_O_ for high opacity. This indicates that correctly perceiving the target when it is strongly masked reflects some common ability across faces and non-face object categories (see last two lines of table 4).

Next we used the bivariate correlations among both orientations of the same test condition to test whether performance is solely explained by their common variance. Decomposing the proportion of reliable variance into a predicted and a residual component, $r_{\max}^{2}=r^{2}+r_{\mathrm{res}}^{2}$, we tested the residual component with an *F*- statistic, i.e. $F=r_{\mathrm{res}}^{2}(N-2)/(1-r_{\mathrm{res}}^{2})$ [57]. Results (table 5) revealed that a significant proportion of reliable residual variance remained in each test condition, whereby the proportions of explained and residual variance were nearly alike. Proportions of residual variance tended to be larger for the tests with higher reliability (CFMT and CCP), while they were smaller for the less reliable SPP.

**Table 5.** Pearson product-moment correlations of upright and inverted orientation for each test condition. The table shows the correlation coefficient, *r*, the theoretical maximum correlation based on test condition reliability in either orientation, *r*_max_, proportion of common variance, *r*^2^, theoretical maximum proportion of common variance, $r_{\max}^{2}$, proportion of residual variance, $r_{\mathrm{res}}^{2}$, its *F*-statistic and significance level, *p*.

test	condition	*r*	*r*_max_	*r*^2^	$r_{\max}^{2}$	$r_{\mathrm{res}}^{2}$	*F*_1,296_	*p*
CFMT	all	0.50	0.78	0.25	0.61	0.36	169.5	<0.001
CCP	congruent	0.52	0.86	0.27	0.74	0.47	265.3	<0.001
	incongruent	0.58	0.80	0.33	0.64	0.31	130.8	<0.001
SPP	face-LO	0.33	0.36	0.11	0.13	0.02	5.42	<0.05
	face-HO	0.22	0.36	0.05	0.13	0.08	25.9	<0.001
	scene-LO	0.50	0.58	0.25	0.33	0.08	27.1	<0.001
	scene-HO	0.44	0.56	0.20	0.32	0.12	40.9	<0.001

Hence, results showed that not all of the reliable variance for upright stimuli could be predicted by the reliable variance for inverted stimuli in the same test condition. The strong correlations across orientation clearly suggest a common route for both orientations. However, significant proportions of residual variance indicate that additional orientation-specific components enter.

In the next steps, we analysed whether common variance across test conditions was orientation-invariant.

#### 3.5.2. Multiple regression results

Multiple regression was used to predict the CFMT by the CCP, the SPP_F_ and the SPP_O_. To prove whether equal predictability results when criterion and predictors have same or opposite orientation, we ran separate analyses for each combination of predictor and criterion orientations, resulting in four analyses. Table 6 summarizes the overall results. All four analyses revealed highly significant predictability of the CFMT by the test batteries. The multiple correlation reached by the predictor batteries was $\bar{R}=0.379$ on average, obtained by using Fisher *Z* transforms of the multiple correlation coefficients. Predictability in the four orientation configurations was alike, showing only marginal deviation from the average and largely overlapping confidence intervals. The largest multiple correlation was observed for predicting the CFMT in inverted presentation from CCP and SPP predictors in upright configuration.

**Table 6.** Multiple regression results for estimating the CFMT from test batteries formed by CCP, SPP_F_ and SPP_O_ conditions in definite orientations. The table shows the orientation conditions for criterion and predictors, multiple correlation coefficient, *R*, model and residual sums of squares and degrees of freedom, *SSQ* and d.f., *F* ratio, significance level, *p* and the 95% confidence interval of the multiple correlation coefficient, CI.

criterion	predictors	*R*	SSQ_*m*_	d.f._*m*_	SSQ_*e*_	d.f._*e*_	*F*	*p*	CI(*R*)
upright	upright	0.37	0.64	6	4.11	291	7.56	<0.001	[0.27,0.46]
upright	inverted	0.33	0.52	6	4.23	291	5.96	<0.001	[0.23,0.43]
inverted	upright	0.42	0.46	6	2.22	291	10.07	<0.001	[0.32,0.51]
inverted	inverted	0.40	0.44	6	2.24	291	9.48	<0.001	[0.31,0.50]

Table 7 shows the detailed results for the four test batteries, allowing identification of the most valid predictors for the CFMT. Across all four analyses results were practically coincident. The CCP in congruent condition was the best predictor of the CFMT, followed by the SPP_F_ in the high opacity condition. Only in one case did the low opacity condition of the SPP_F_ reach significant contributions to the CFMT criterion. The CCP in incongruent condition and all conditions of the SPP_O_ clearly failed to contribute to valid CFMT predictions. Importantly, whether a test condition was a valid predictor or not did not depend on the orientation of criterion or predictors. Hence, the results from multiple regression analysis strongly indicate that just the nature of the task underlying the predictors defined the common variance with the CFMT, but not their orientation.

**Table 7.** Multiple regression results for estimating the CFMT from test batteries formed by CCP, SPP_F_ and SPP_O_ conditions in definite orientations. The table shows predictor with condition, standardized regression coefficient, *b*, its standard error, *s*_*b*_, product moment correlation of predictor and criterion, *r*_*c*_, *t*-statistic with degrees of freedom and significance level, *p*. Significant regression coefficients are printed boldface.

Pred	Cond	*b*	*s*_*b*_	*r*_*c*_	*t*_291_	*p*	*b*	*s*_*b*_	*r*_*c*_	*t*_291_	*p*
		upright criterion and upright predictors					upright criterion and inverted predictors				
CCP	CC	**0.26**	0.057	0.28	4.51	<0.001	**0.22**	0.058	0.27	3.85	<0.001
CCP	IC	0.02	0.057	0.10	0.38	0.71	0.03	0.058	0.13	0.58	0.562
SPP_F_	LO	**0.15**	0.058	0.19	2.60	<0.01	0.07	0.061	0.16	1.06	0.292
SPP_F_	HO	**0.15**	0.058	0.22	2.50	<0.01	**0.15**	0.062	0.21	2.39	<0.02
SPP_O_	LO	−0.01	0.062	0.04	−0.16	0.871	0.06	0.063	0.11	0.97	0.334
SPP_O_	HO	0.01	0.066	0.11	0.18	0.854	−0.05	0.063	0.04	−0.74	0.461
		inverted criterion and upright predictors					inverted criterion and inverted predictors				
CCP	CC	**0.31**	0.056	0.35	5.60	<0.001	**0.23**	0.056	0.27	4.08	<0.001
CCP	IC	0.03	0.055	0.13	0.56	0.578	0.024	0.057	0.12	0.43	0.668
SPP_F_	LO	0.06	0.056	0.12	1.10	0.274	**0.17**	0.059	0.27	2.83	<0.01
SPP_F_	HO	**0.16**	0.057	0.24	2.83	<0.01	**0.17**	0.060	0.27	2.75	<0.01
SPP_O_	LO	−0.04	0.061	0.06	−0.59	0.556	−0.09	0.061	0.03	−1.42	0.158
SPP_O_	HO	0.09	0.064	0.17	1.46	0.144	0.08	0.061	0.13	1.33	0.183

To further validate this result, we used bivariate regression to predict the scores for upright from the scores for inverted stimuli for each test condition, and analysed the predicted scores, $\hat{z}$, as well as the residual scores, *z*_*e*_, with multiple regression. Note that $\hat{z}$ are the scores expected from orientation-invariant processing, since they capture the variance which is shared by both orientations. The *z*_*e*_ scores are the scores which remain unexplained by this assumption, thus they capture the orientation-specific variance. If all common test variance is orientation-unspecific, then predicting the CFMT $\hat{z}$ scores by a multiple regression battery of $\hat{z}$ scores from the other tests identifies the significant predictors, while predicting CFMT *z*_*e*_ scores should result in a non-significant prediction battery. Multiple regression results exactly complied with this prediction. For $\hat{z}$ scores we obtained a highly significant multiple regression battery (*R* = 0.40, *F*_6,291_ = 9.48, *p* < 0.001), while the battery for *z*_*e*_ scores was not significant (*R* = 0.18, *F*_6,291_ = 9.48, *p* = 0.148). Detailed results (table 8) substantiated that the CCP in congruent condition was the best predictor of the CFMT, followed by both opacity conditions of the SPP_F_.

**Table 8.** Multiple regression results for estimating the CFMT from test batteries formed by CCP, SPP_F_ and SPP_O_ conditions, using scores predicted from common orientation processing in each test ($\hat{z}$, left columns), and the residual scores that are unexplained by this assumption (*z*_*e*_, right columns). The table shows predictor with condition, standardized regression coefficient, *b*, its standard error, *s*_*b*_, product moment correlation of predictor and criterion, *r*_*c*_, *t*-statistic with degrees of freedom, and significance level, *p*. Significant regression coefficients are printed boldface.

		$\hat{z}$ scores					*z*_*e*_ scores				
Pred	Cond	*b*	*s*_*b*_	*r*_*c*_	*t*_291_	*p*	*b*	*s*_*b*_	*r*_*c*_	*t*_291_	*p*
CCP	CC	**0.23**	0.056	0.27	4.08	<0.001	0.06	0.058	0.05	1.00	0.314
CCP	IC	0.02	0.057	0.12	0.43	0.668	−0.01	0.058	−0.01	−0.15	0.881
SPP_F_	LO	**0.17**	0.059	0.27	2.83	<0.01	**0.13**	0.059	0.14	2.25	<0.05
SPP_F_	HO	**0.16**	0.060	0.27	2.75	<0.01	0.07	0.059	0.09	1.26	0.207
SPP_O_	LO	−0.09	0.061	0.03	−1.42	0.158	−0.04	0.059	−0.03	−0.75	0.453
SPP_O_	HO	0.08	0.061	0.13	1.33	0.183	0.02	0.060	0.05	0.35	0.724

#### 3.5.3. Principal components

Principal component decomposition was calculated to reveal the factor structure of all seven test conditions in the two orientations (14 variables). This analysis revealed a typical exponential decay of the Eigenvalues, and identified four factors with Eigenvalue above 1 (figure 3). To verify a four factors solution for the true common variance components, we additionally used Horn’s modification of the factor-scree test [58]. Generating 14 normally distributed and independent random variables with the same set size as the dataset (*N* = 298) and applying PCA yielded a linear Eigenvalue course (see grey values and straight line in figure 3). Only the first four factors of the dataset had Eigenvalues above the Eigenvalues of the random variables (Horn criterion). Together, these four factors extracted 58.5% of test variance from the correlation matrix. We rotated with a Varimax standardized procedure to achieve a simple structure in the factor loadings pattern. The results of this solution are shown in table 9. A clear-cut simple structure was obtained, featuring four factors which accounted for approximately 15% of the total test variance each. The first factor represented shared variance of the CFMT (40.4%) and the CCP in congruent condition (52.9%). The second factor was specific for the SPP_O_ (96.3%) and the third factor was specific for the CCP in incongruent condition (88.6%). Neither of them had common variance with the CFMT nor with other tests. The fourth factor was specific for the SPP_F_ (82.6%), but also represented common variance with the CFMT (9.9%). These results strongly correspond to the result obtained in the multiple regression analyses. The CCP in congruent condition and, to minor degrees, the SPP_F_ shared distinct and independent (orthogonal) common variance components with the CFMT. The CCP in incongruent condition and the SPP_O_ did not share common variance with the CFMT, nor with other tests,^7^ representing specific abilities with no link to others. A further very striking result of the principal component analysis is that, if there were strong factor loadings, they had equal magnitudes in upright and in inverted orientations. The factor loadings uniquely reflect orientation-invariance of specific test variance and of common test variance. The pattern of factor loadings consistently showed that both orientations of the same test conditions always loaded together on the same factors. There was no sign of orientation-specific variance in the factor solution. The orientation-invariant factor solution was validated against potential metric quality constraints by analysing Spearman rank correlations, which yielded practically the same factor loadings pattern (see appendix B).

**Table 9.** Factor loadings of all experimental conditions in both orientations for a four factor solution (λ > 1), after varimax standardized rotation. The table shows the factor loadings, explained variance in Eigenvalue units, proportion of explained variance of each factor (%), proportion of test-specific variance of the test with dominant loadings on the factor (%), proportion of variance of the CFMT on the factor (%) and the sum of test-specific and CFMT variance on the factor. Factor loadings larger than 0.5 are printed boldface.

test	condition	orientation	*F*_1_	*F*_2_	*F*_3_	*F*_4_
CFMT	—	upr	**0.63**	−0.01	−0.02	0.29
CFMT	—	inv	**0.67**	0.00	−0.05	0.35
CCP	CC	upr	**0.77**	0.08	0.21	−0.04
CCP	CC	inv	**0.72**	0.12	0.20	−0.05
CCP	IC	upr	0.13	0.08	**0.85**	0.01
CCP	IC	inv	0.15	0.06	**0.85**	0.15
SPP_F_	LO	upr	−0.10	0.09	0.20	**0.56**
SPP_F_	LO	inv	0.17	0.12	−0.13	**0.71**
SPP_F_	HO	upr	0.18	0.17	−0.03	**0.66**
SPP_F_	HO	inv	0.14	0.05	0.14	**0.68**
SPP_O_	LO	upr	0.05	**0.78**	−0.02	−0.03
SPP_O_	LO	inv	0.07	**0.76**	0.11	0.06
SPP_O_	HO	upr	0.02	**0.75**	0.10	0.31
SPP_O_	HO	inv	0.05	**0.73**	0.01	0.17
explained Var			2.09	2.38	1.64	2.08
total (%)			15.0	17.0	11.7	14.9
test-specific (%)			52.9	96.3	88.6	82.6
CFMT (%)			40.4	< 0.1	0.2	9.9
test+CFMT (%)			93.3	96.3	88.8	92.5

### 3.6. Discussion of individual differences results

Both regression and principal component analysis showed that the CFMT shared common variance with the CCP in congruent condition and with the SPP_F_, while the latter two did not share common variance. Thus, performance in the CCP (congruent), measuring face identity perception across swapped $\frac{3}{4}$ views and in the SPP_F_, measuring object-related attention to faces, likely concern different and independent abilities, both contributing to the face recognition ability. In either orientation, the congruent condition of the CCP predicted face recognition, while the incongruent condition failed to do so. This is clear evidence that task success in congruent and incongruent condition of the CCP rest on distinct attentional strategies, thus validating the assumptions of the paradigm.^8^ Precise focus on just the inner face parts, which warrants success in the incongruent condition, is unrelated to CFMT performance, while attending the whole facial appearance including its external features [59,60] improves face recognition.

These results correspond to recent findings reported for the composite face paradigm [45]. For this paradigm, authors reported the largest correlation with the CFMT in the aligned-congruent condition (*r* = 0.5) and the smallest correlation in the aligned-incongruent condition (*r* = 0.21, see [45], table 1). These results might suggest that the ability to precisely keep focus on facial target parts is less important for face recognition than the ability to assess face identity using a more global attentional strategy, since this allows for a richer and deeper face encoding particularly for changing views [28,61–63]. However, it was pointed out [37,44] that the CFMT with its constant repetition of the six target faces has a clear learning format, and tests which use repetition of faces or face parts could correlate with the CFMT due to similar learning formats. Indeed, this potential confounding concerns the CCP, since the composites in the CCP stem from only four base faces.^9^

Second, we found that face-selective attention under conditions of visual competition (SPP_F_) explains face recognition, while scene-selective attention SPP_O_ does not. This finding is in line with the strong category specificity of face recognition [29,36,37]. Since the gender categorization task favoured usage of whole face-based strategies [64], this corresponds to the finding that only the congruent condition of the CCP shared variance with the CFMT. In the SPP, the same face appeared eight times (four times as a distracter and four times as the target), and presentation time was quite long (2.5 s). This could have enabled face learning. Indeed, as a result of repetition, there were adaptation effects in the FFA and the PPA (parahippocampal place area), which were shown to correlate with object recognition tasks administered after the SPP [51]. These findings indicated a relationship between intact early attentional selection mechanisms, selective and more strongly tuned activation of stimulus category-specific extrastriate brain areas [52], and better encoding. In line with these results, our findings corroborate that observers who better attended faces and more efficiently suppressed scenes also showed better face recognition performance. The independence of SPP_F_ and the CCP (both conditions) showed that this object-related attentional ability is clearly distinguished from the ability to attend faces with different spatial foci.

## 4. General discussion

Testing face recognition, face perception and object-based attention to faces and scenes with a comprehensive test battery revealed consistent orientation-invariance in all patterns of associations and dissociations. If test conditions shared common variance, they shared it within the same and across different orientations. Factor loadings of test conditions on common or unique factors, which accounted for 58.5% of the total test variance, were always equivalent in magnitude for either orientation. Prediction of the CFMT succeeded equally well both within same orientations and across them, and the best CFMT predictor battery was validated by the scores predicted from a common mechanism for upright and inverted stimuli. Thus, individual difference analysis indicated the absence of orientation-specific correlation structures. There was no evidence for task-general processing specific for either orientation, and therefore, there was also no support for the claim that inverted faces rely on a task-general, part-driven processing mode, while upright stimuli are handled by globally face-tuned and ‘holistic’ mechanisms [2,9].

On the other hand, an orientation-general route could not completely explain all reliable test condition variance, which indeed indicated involvement of orientation-specific components. This finding is in agreement with earlier observations from a smaller sample with *N* = 77 participants [25].^10^ Since we found that the correlations within and between tests were strictly orientation-invariant, orientation-specific processing is limited to the single test conditions. Therefore, our findings support a weaker claim of orientation-invariance, which permits involvement of orientation-specific processes, but assumes that all shared processes within and across tests are orientation-invariant. Particularly, we found evidence that only orientation-invariant processing links face recognition to face perception and face-selective attention. These results are plausible in the light of current findings, which corroborate that the same face-selective brain areas are involved in different face processing tasks [13,18,65], while inverted faces engage mechanisms in face- and non-face object selective areas [4,14,66]. Results of a recent study [24] corroborated quantitative orientation-based modulation in mechanisms of the basic face network (early visual cortex, OFA, FFA and inferior frontal gyrus), which are sensitive to faces in either orientation, while only upright faces induced inhibition in the general object-tuned lateral occipital complex (LOC), and only inverted faces caused activation in the intraparietal sulcus (IPS), which is associated with visual working memory [67]. These results provide evidence both for quantitative changes of activity in common, orientation-general mechanisms along the central face processing route and broader involvement of generally object-tuned areas for inverted faces.

What are the implications for the debate about potential qualitative changes of face processing due to picture-plane inversion? Assuming that performance in a given test condition relies on task-related and condition-specific processes, our findings imply only quantitative changes in the more general, task-related processes and putative qualitative changes in the components that are added to handle a specific test condition. Individual differences analysis cannot further resolve the latter, because it rests on common variance. Hence, the condition-specific processes which contingently change with stimulus orientation could be strong unique components, or fall into several scattered and inconsiderable ones.

The single test results of this study, however, do not support major qualitative changes due to picture-plane inversion. There were strong interactions of congruency (CCP), and opacity (SPP), with orientation. However, closer analysis showed that the effect of external context was diminished for inverted faces, but it remained substantial. Likewise, we found that attentional competition among target object and masker was stronger for inverted presentation, indicating that upright stimuli yielded more robust object representations. Yet, opacity effects were strong in either orientation, indicating that inversion did not affect the kind of attentional interaction. Hence, the experimental results of the tests used here add to a line of evidence showing that, albeit with reduced efficiency, the way observers handle task demands does not change qualitatively with stimulus inversion [20–22].^11^

The finding of orientation-invariant correlation structures, though, could derive from the specific test selection. We tested face recognition, face identity matching with congruent and incongruent external features, and object-based attention to faces and scenes, while sensitivity to facial feature spacing [25,70,71], matching face parts and wholes [10] and face identity matching with internal feature composites [11,72] were not tested. Since the discussion about potential orientation-specific processing frequently referred to experimental results from spacing and composite face tasks [2], it would be highly desirable to have individual difference results from these paradigms, based on a large sample. This could clarify whether there is indeed orientation-invariance of common variance in a wide range of tasks for key aspects of face processing. A further limitation is that only one test for non-face objects was included. Recent research showed the importance of using several different object categories, since the distinctiveness of faces is not necessarily greater than the distinctiveness of other object categories (e.g. cars, [36]), which could be uncovered only by using objects from many different classes, as in the Vanderbilt Expertise Test [37]. Using a wide range of object classes and controlling for expertise, a domain-general object ability could be revealed, which proved to be rather independent from general intelligence and other cognitive and perceptual skills [73,74]. With such a comprehensive approach, it would be challenging to show that domain-general abilities are independent of stimulus orientation. The present study was a first attempt to reveal whether and how stimulus orientation modifies the pattern of associations and dissociations in face processing tasks. Further experimentation in the framework of the individual differences approach is necessary to gauge whether orientation-invariance in the shared variance components of face- and non-face related tasks can claim generality.

## 5. Conclusion

Studying face recognition, face perception and face-selective attention with a comprehensive test battery has revealed orientation-invariance of common variance within and among these domains of face-processing. Face inversion impaired performance seriously, but did not change the specific patterns of associations and dissociations within and across tests. This suggests that process-specific, but orientation-general mechanisms drove common variance in the face related tasks. Our findings add to a line of research which shows that, after abstracting from the performance decline for inverted presentation, key features of face processing, like spatial frequency tuning or cue usage in face image classification, are orientation-invariant.

## Supplementary Material

Scatter Plots

## Footnotes

1 Authors found one exception to the robust simple structure in the factor loadings pattern. The spacing condition for upright faces had a high loading on the spacing factor, but also loaded on the parts factor, and the same dual loadings pattern was found for the part condition, which had a high loading on the parts factor but also on the spacing factor. This association of part and spacing conditions only for upright faces was reflected by zero-order correlations, which let authors conclude that part and spacing are jointly processed, but only for upright faces.

2 A major problem in this line of research was that derived measures of holistic processing suffered from reduced reliability [43,45,47]. Richler and colleagues introduced a new variety of the composite face test (VHPT-F), which yielded a composite effect measure with much improved reliability [43]. Testing in several larger samples showed no correlation with face recognition, tested with the CFMT. Further tests with the standard composite face paradigm using smaller and larger face sets revealed significant correlations with the CFMT only for small face sets. This indicated that face learning was the critical link between the CFMT and the composite effect [44].

3 Since this paradigm is a congruency paradigm, it allows using a congruency effect measure to index holistic processing [48]. Please note that predicting face recognition from derived measures of holistic processing, and their methodological constraints and issues, is beyond the scope of this study.

4 Opacity effects were larger for attending faces compared to attending scenes on the proportion correct scale (figure 2), but statistical effects were larger for attending scenes due to the very small standard error in this task.

5 In the factor analysis literature, the common variance components denote the components that can be separated from unreliable, spurious components in the solution of the factor problem (determination of the number of factors) [56].

6 Note that if there are strong loadings of test conditions in both orientations on the same factor, this implies that there are substantial correlations across orientations.

7 One exception is a loading of SPP_O_ on *F*_4_, reflecting some common variance of SPP_O_ and SPP_F_ in HO (see also zero-order correlations).

8 It is shown in appendix A that performance in the congruent condition controlled for performance in the incongruent condition by regressing out the influence of the incongruent condition [45] predicts the CFMT fairly like the congruent condition of the CCP alone does. This shows that the congruent condition already contains the relevant criterion variance, while the variance components contained in the incongruent condition are irrelevant.

9 In the CCP, a definite composite face is selected from the 16 possible instances only ones. Ones selected, it has to be used in 4 trials to fulfill the constraints of the complete design (see [48, fig. 1]). The face parts for creating composites occur much more often, enabling the observers to get familiar with them across trials.

10 Authors obtained largest correlations within the same test conditions, but across orientation, which were about *r* ≈ 0.5 ([25, table 2]). This exactly complies with our findings. Their experimental test had reliability of *r*_*tt*_ ≈ 0.8 for spacing and part conditions ([25], table 2), which corresponds to CFMT and CCP reliabilities. Hence, as observed here, reliable variance for upright stimuli could not fully be explained by reliable variance for inverted ones.

11 Another strong observation in this line of evidence comes from the composite paradigm, in the variety of the complete design [50,68]. The alignment × congruency interaction is disordinal, showing a pronounced scissors pattern when ‘aligned’ and ‘misaligned’ are the categories on the *x*-axis and performance for congruent and incongruent condition is shown by two lines (e.g. [69]). This disordinal interaction means that the misaligned condition improves performance for incongruent composites and worsens performance for congruent ones. For inverted presentation, the slopes of the two legs are somewhat attenuated, but the interaction is still strictly disordinal (e.g. [23]). A change of the interaction type with inversion has so far not been reported in the literature.

## Data accessibility

The dataset of this study is accessible in the Dryad Open Data repository: https://doi.org/10.5061/dryad.4dv51ps [75].

## Appendix A. Individual differences results for the congruency effect of the CCP

Because the performance difference for congruent and incongruent conditions (congruence effect, CE) is used as an index for the contextual effect of the external features in group-level studies [12], we also report results from individual differences for this measure. Congruent and incongruent conditions were modestly intercorrelated (upright: *r*(CC, IC) = 0.23; inverted: *r*(CC, IC) = 0.25). Based on the internal consistency of the CCP (table 2), the reliability (*α*) of the CE was estimated using Guilford’s standard formula for the reliability of differences, *r*_dd_ = (*r*_tt_(CC) + *r*_tt_(IC))/2(1 − *r*(CC, IC)), which showed *r*_dd_ = 0.81 for upright and *r*_dd_ = 0.74 for inverted stimuli. Correlations of the CE with the CFMT are shown in table 10 (left part). Since it was suggested to estimate correlations with face recognition by correlating the condition of interest (here: congruent condition) after regressing out the effects of the control condition (here: incongruent condition) [45], we also report results for this method (table 10, right part). Results showed that the congruency effect, calculated as a difference measure, did not correlate with the CFMT. Based on the regression measure, we found highly significant correlations, which fairly well agreed with the correlations of the congruent condition with the CFMT (table 4). Also the correlations across orientation nearly coincided. These results support that the congruent condition of the CCP contains the variation of interest, while congruent and incongruent condition capture distinct attentional strategies for judging face identity. These results closely correspond to the results from multiple regression analysis, which showed that the congruent but not the incongruent condition predicted the CFMT.

**Table 10.** Pearson product-moment correlations for the difference-based (left) and regression-based measure (right) of the contextual effect in the CCP with the CFMT. Non-significant correlations below the critical correlation $r_{\left(5\text{\%}\right)}=0.11$ are printed normal, correlations above the critical correlation $r_{\left(0.1\text{\%}\right)}=0.19$ are printed boldface.

measure		difference			regression		
test		CFMT		CCP	CFMT		CCP
	orient	upr	inv	upr	upr	inv	upr
CCP	upr	0.05	0.06		**0.27**	**0.33**	
CCP	inv	0.08	0.09	**0.44**	**0.24**	**0.25**	**0.47**

## Appendix B. PCA results for Spearman-rank correlations

To check whether the specific factor loadings pattern shown in table 9 depended on the metric quality of the experimental tests, we calculated Spearman rank correlations of the data and analysed the correlation matrix with PCA. Again four factors with Eigenvalue above 1 were obtained, which together explained 53% of test variance (table 11). After varimax standardized rotation, we obtained practically the same loadings pattern as for Pearson product-moment correlations, with but slightly reduced explained variance per factor. Concerning *F*_1_, *F*_3_ and *F*_4_ results were nearly identical for both correlations methods, while *F*_2_ explained 4.25% less variance, mostly from SPP_O_ contributions. This may indicate reduced metric quality of the SPP_O_, which had relatively low difficulty, and its variance range was therefore restricted. Overall, however, the comparison shows that the particular simple structure reflecting a highly orientation-invariant loadings pattern on common and test-specific factors did not depend on potential metric quality constraints.

**Table 11.** Factor loadings for a four factors solutions (λ > 1) after varimax standardized rotation, based on Spearman rank correlations of all experimental conditions in both orientations. Presentations conventions as in table 9.

test	condition	orientation	*F*_1_	*F*_2_	*F*_3_	*F*_4_
CFMT	—	upr	**0.64**	−0.03	−0.02	0.28
CFMT	—	inv	**0.65**	−0.03	−0.02	0.35
CCP	CC	upr	**0.74**	0.15	0.24	−0.04
CCP	CC	inv	**0.72**	0.09	0.19	−0.03
CCP	IC	upr	0.14	0.05	**0.83**	−0.03
CCP	IC	inv	0.17	0.07	**0.83**	0.14
SPP_F_	LO	upr	−0.12	0.08	0.25	**0.53**
SPP_F_	LO	inv	0.20	0.13	−0.18	**0.68**
SPP_F_	HO	upr	0.18	0.16	−0.03	**0.61**
SPP_F_	HO	inv	0.15	0.07	0.12	**0.66**
SPP_O_	LO	upr	0.07	**0.67**	−0.09	−0.05
SPP_O_	LO	inv	0.06	**0.67**	0.13	0.04
SPP_O_	HO	upr	−0.05	**0.64**	0.12	0.34
SPP_O_	HO	inv	0.06	**0.62**	0.03	0.22
explained Var			2.07	1.79	1.61	1.95
total %			14.8%	12.8%	11.5%	13.9%
test-specific %			51.7%	94.8%	85.0%	79.9%
CFMT %			40.0%	<0.1%	0.1%	9.7%
Test + CFMT %			93.7%	94.8%	85.1%	89.6%
